# Supplementary material for: Adipose-Derived Mesenchymal Stem Cells Protect Endothelial Cells from Hypoxic Injury by Suppressing Terminal UPR In Vivo and In Vitro
Source: Int J Mol Sci. 2023 Dec 6;24(24):17197. doi: 10.3390/ijms242417197 (PMC10742997; doi:10.3390/ijms242417197)
Supplement: Supplementary file 1 [file ijms-24-17197-s001.zip › ijms-2674646-supplementary.pdf]

Supplementary Materials:

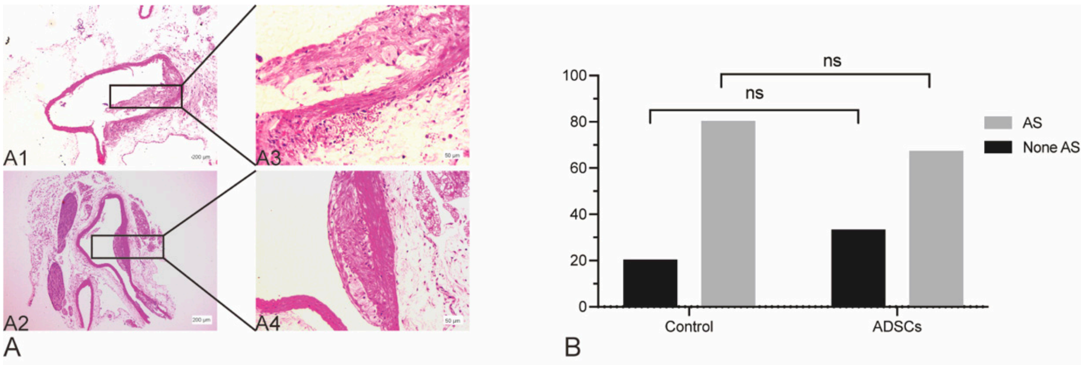

Figure S1.
A: H&E sections of the thoracoabdominal aorta. The mouse aorta cross-section was analyzed by HE staining, where A1 and A2 are the conditions under the 200 μm field of view, and A3 and A4 are the conditions under the 50 μm field, indicating that the mouse aorta has developed atherosclerotic plaques. B: No significant difference was observed between the treatment and the control groups (Two-way ANOVA analysis was performed).

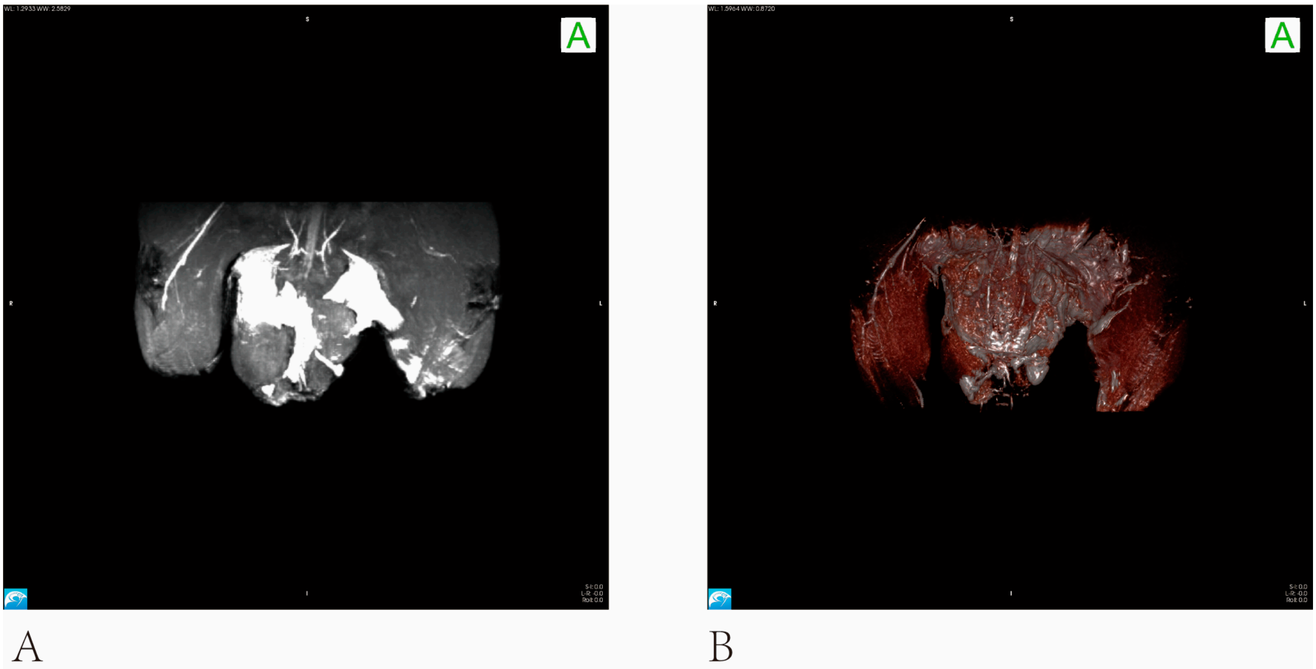

Figure S2: The (A) MRI scan and (B) 3D reconstruction results showed that the arterial blood flow of the right lower extremity had been effectively cut off.

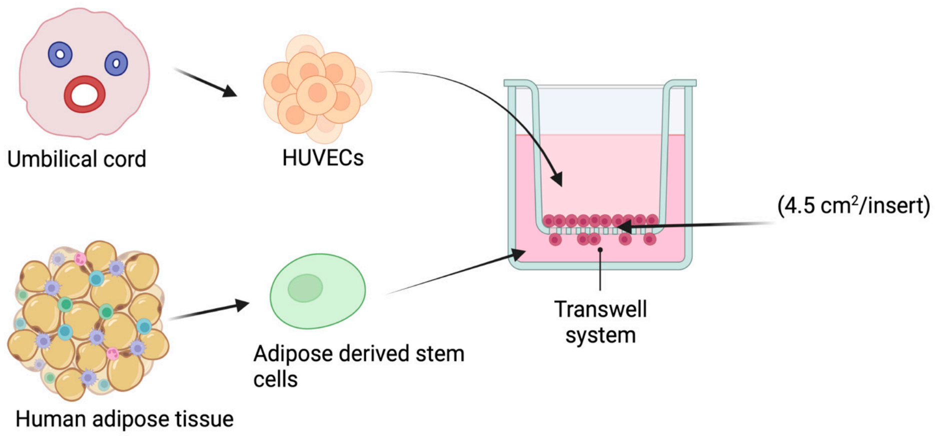

Figure S3. Human umbilical cord vein endothelial cells isolated from human umbilical cord veins and human adipose stem cells isolated from human adipose tissue were co-cultured using the transwell system, in which insert (4.5cm^2) was used. The upper layer was adipose stem cells and the lower layer was endothelial cells.
